# Structural and magnetic depth profiles of magneto-ionic heterostructures beyond the interface limit

**DOI:** 10.1038/ncomms12264

**Published:** 2016-07-22

**Authors:** Dustin A. Gilbert, Alexander J. Grutter, Elke Arenholz, Kai Liu, B. J. Kirby, Julie A. Borchers, Brian B. Maranville

**Affiliations:** 1 NIST Center for Neutron Research, National Institute of Standards and Technology, Gaithersburg, Maryland 20899, USA; 2 Advanced Light Source, Lawrence Berkeley National Laboratory, Berkeley, California 94720, USA; 3 Physics Department, University of California, Davis, California 95616, USA

## Abstract

Electric field control of magnetism provides a promising route towards ultralow power information storage and sensor technologies. The effects of magneto-ionic motion have been prominently featured in the modification of interface characteristics. Here, we demonstrate magnetoelectric coupling moderated by voltage-driven oxygen migration beyond the interface in relatively thick AlO_x_/GdO_x_/Co(15 nm) films. Oxygen migration and Co magnetization are quantitatively mapped with polarized neutron reflectometry under electro-thermal conditioning. The depth-resolved profiles uniquely identify interfacial and bulk behaviours and a semi-reversible control of the magnetization. Magnetometry measurements suggest changes in the microstructure which disrupt long-range ferromagnetic ordering, resulting in an additional magnetically soft phase. X-ray spectroscopy confirms changes in the Co oxidation state, but not in the Gd, suggesting that the GdO_x_ transmits oxygen but does not source or sink it. These results together provide crucial insight into controlling magnetism via magneto-ionic motion, both at interfaces and throughout the bulk of the films.

## Abstract (web summary)

Mechanisms allowing electrical manipulation of magnetic material possess potential applications in low power memory and sensor technologies. Here, the authors demonstrate the control of magnetic characteristics via voltage-driven migration of oxygen across a GdOx/Co interface, well into the bulk of the cobalt.

## Graphical abstract

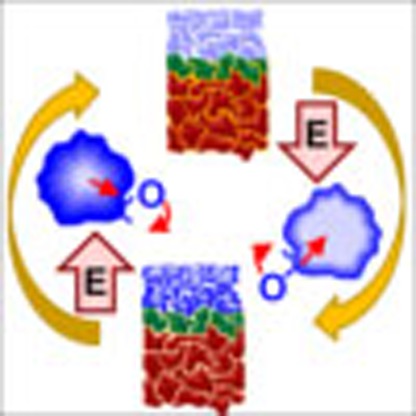

With exciting potential for energy-efficiency and new functionalities in hybrid magnetoelectric devices, voltage control of magnetism is currently the focus of intense investigations^1^,^2^,^3^,^4^,^5^. Traditional spintronic architectures use electron spin for information storage and transmission, for example, utilizing the giant^6^,^7^ or tunnelling^8^,^9^ magnetoresistance, spin transfer torque^10^,^11^,^12^ or the spin Hall effects^13^,^14^. Recently, oxygen ion migration has been demonstrated as an effective approach to control magnetic properties^15^,^16^,^17^,^18^,^19^,^20^. These magneto-ionic architectures use electrical or chemically motivated effects to control oxygen ion distributions, thus achieving tunability of interface and bulk properties. A combined heating and electric field (electro-thermal (E+T)) conditioning sequence has been demonstrated in GdO_x_/Co Hall bar structures to drive oxygen from an oxide film into a neighbouring ferromagnetic metal layer^15^,^16^,^17^,^18^ and directly tailor the interface chemistry. In so doing, magnetic anisotropy and saturation magnetization at the oxide/ferromagnet interface are effectively controlled by the electric field, even reversibly. These architectures offer advantages over alternative spintronic designs including minimal energy dissipation due to, for example, Joule heating and an expected non-volatility. The most prominent effect demonstrated so far is the modification of the interface—indeed the ultrathin Co films in these studies are less than five monolayers thick, and are consequently dominated by the interfacial properties. Considering the strong reduction potential of gadolinium^21^ and relatively good chemical stability of cobalt oxide^22^, the observed reversibility cannot be explained by simple enthalpy considerations alone, suggesting that the bulk and interfacial behaviours may be different. However, probing the magneto-ionic motion in the bulk of the film is intrinsically challenging.

In this work, we report direct mapping of structural and magnetic depth profiles in voltage-controlled magneto-ionic heterostructures with relatively thick (15 nm) films of Co, and demonstrate that the electrically induced oxygen migration extends far beyond the interface. Using polarized neutron reflectometry (PNR), the electrically induced oxygen migration is directly mapped in the nuclear scattering length density (SLD). In contrast to previous results, PNR is a large-area, non-destructive measurement technique which simultaneously maps changes in oxygen distribution and magnetization. PNR depth profiles show that the electric field drives oxygen deep into the Co film (>10 nm), reducing the magnetization by >80% at the interface and 38% in the bulk. After reversing the polarity of the applied electric field, the magnetization recovers to 92% of the original value throughout the Co layer. Thus interface and bulk-like behaviours are demonstrated to be different. Using the first-order reversal curve (FORC) technique, we identify two distinct magnetic phases in the post-conditioned sample. This behaviour differs significantly from that of the as-grown state, which exhibits a single magnetic phase, suggesting that the oxygen migration significantly alters the magnetic characteristics by impeding inter-granular coupling. Control experiments performed in the absence of an electric field reveal that annealing alone causes much smaller structural and magnetic changes to the Co film, distinguishing the roles of thermal conditioning sequences from E+T ones. X-ray absorption (XA) spectroscopy and X-ray magnetic circular dichroism (XMCD) confirm the role of the electric field in recovering the magnetization and cobalt oxidation states. XA performed on the GdO_x_ shows that the Gd oxidation state is invariant, suggesting that the Gd transmits the oxygen rather than surrendering it.

## Results

### Polarized neutron reflectometry

**Figure 1:**
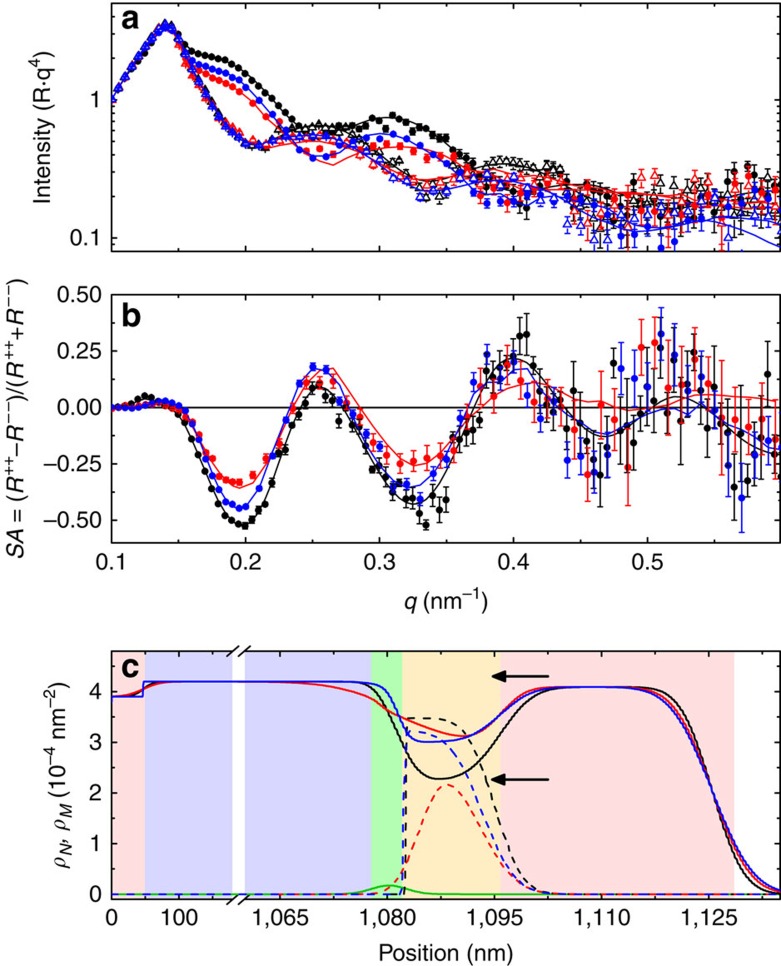
PNR results of E+T-conditioned sample. (**a**) Fitted PNR data, with *R*^*++*^ (*R*^*−−*^) identified by solid circles (open triangles), scaled by *q*^4^ and (**b**) spin asymmetry for the sample as-grown and after E+T conditioning. (**c**) Depth-dependent real and imaginary nuclear SLD (*ρ*_*N*_ and *ρ*_*imag*_), and magnetic SLD (*ρ*_*M*_) extracted from the PNR. In all panels black, red and blue lines identify the as-grown, +40 V conditioned, and +/−40 V conditioned samples, respectively. In **c** the solid and dashed lines identify *ρ*_*N*_ and *ρ*_*M*_, respectively, and the green line identifies *ρ*_*imag*_. Background colours in **c** represent (red) Pd, (purple) AlO_x_, (green) GdO_x_ and (yellow) Co, respectively. In **a** and **b** the experimental data are shown as symbols, and the lines are fits corresponding to the depth profile shown in **c**. Error bars in **a** and **b** correspond to ±1s.d.; error bars for **c** are shown in Supplementary Fig. 2. Arrows in **a** and **b** identify the bulk *ρ*_*N*_ for Co (2.25 × 10^−4^ nm^−2^) and CoO (4.29 × 10^−4^ nm^−2^).

Thin film samples of Si/Pd (50 nm)/AlO_x_ (1 μm)/GdO_x_ (2 nm)/Co (15 nm)/Pd (20 nm) were grown by sputtering and evaporation. The thin GdO_x_ layer is crucial, and experiments performed without the 2 nm GdO_x_ layer were unsuccessful. In contrast to previous studies, this thickness of Co is expected to give rise exclusively to an in-plane magnetic easy axis. The samples were E+T conditioned by heating the sample to 230 °C and applying +40 V to the top Pd film, while the buried Pd film was held at ground for 15 min; the reverse treatment applied −40 V to the top contact, also at 230 °C for 15 min (see the ‘Methods' section). PNR measurements of the as-grown sample and each conditioned state are shown in Fig. 1a. The *R*^*++*^ and *R*^*−−*^ reflectivities show sensitivity to the nuclear and magnetic depth profiles, evident by spin-dependent oscillations. The difference in the *R*^*++*^ and *R*^*−−*^ is approximately proportional to the quotient of the magnetization and the nuclear SLD (see the ‘Methods' section). Thus, the magnetic contribution to the data is highlighted by plotting the spin asymmetry (*SA*=(*R*^*++*^−*R*^*−−*^)/(*R*^*++*^+*R*^*−−*^)), as shown in Fig. 1b. The oscillation amplitude first decreases after conditioning in +40 V then increases after conditioning in −40 V, suggesting a decrease of the saturation magnetization, *M*_*S*_ and/or a change in the structure, followed by a partial recovery towards the initial state. The nuclear and magnetic depth profiles from the converged model, shown in Fig. 1c, confirm these trends. Figure 1 is reconstructed into individual panels in Supplementary Fig. 1 to aid in visualization. The SLD profiles are a cross-sectional average of the nuclear and magnetic SLD at particular depths due to the neutron coherence distribution^23^ and, as such, measure the average contribution of all atoms at that depth.

The extracted depth profile of the as-grown sample accurately reproduces the designed structure, both in terms of thickness and nuclear SLD, *ρ*_*N*_. Our fits show excellent agreement between the measured and expected *ρ*_*N*_ values of Co (2.27 × 10^−4^ nm^−2^), Pd (4.02 × 10^−4^ nm^−2^), and GdO_x_ (2.74 × 10^−4^ nm^−2^) (refs 24, 25). However, the measured SLD of the thick AlO_x_ base layer is substantially lower than the expected bulk value (5.67 × 10^−4^ nm^−2^), suggesting the presence of significant voids or an oxygen-deficient stoichiometry. The GdO_x_—a neutron absorber—can be identified explicitly by the imaginary SLD in Fig. 1c. After conditioning the sample at +40 V, the nuclear SLD of the Co layer, *ρ*_*N*_^*Co*^, increases by 34%, approaching that of CoO (4.29 × 10^−4^ nm^−2^). Simultaneously, the measured GdO_x_/Co interface becomes much broader, increasing from a width of 3.3 to >10 nm and extending well into the AlO_x_. These results are consistent with the electric field removing oxygen from the GdO_x_ and AlO_x_, decreasing their SLD, and depositing it into the Co, increasing its SLD and resulting in an apparent broadening of the interface. After switching the voltage polarity to −40 V, the GdO_x_/Co interface width is reduced to 1.9 nm and *ρ*_*N*_^*Co*^ decreases, demonstrating an induced migration of oxygen from the CoO_x_ into and through the GdO_x_. The recovery of *ρ*_*N*_^*Co*^ occurs predominantly within the 10 nm nearest the GdO_x_ interface, while the top 5 nm, near the Co/Pd interface, remains unchanged from the +40 V conditioned state. Thus, we observe oxygen ion migration throughout the thickness of the Co layer, but reversibly only within the 10 nm closest to the GdO_x_/Co interface. However, at the GdO_x_ interface after this second conditioning, *ρ*_*N*_^*Co*^ is still 32% larger than the as-grown sample, demonstrating that the oxygen migration is only semi-reversible. The uncertainty associated with the presented fits is shown in Supplementary Fig. 2, and discussed in Supplementary Note 1, and is much smaller than the changes between conditioning steps.

Trends in the magnetic depth profile (dashed lines in Fig. 1c) agree with those observed in the nuclear profile. Specifically, the as-grown sample has a sharp step-function like GdO_x_/Co (magnetic) interface and strong magnetic scattering, as indicated by its large magnetic SLD, *ρ*_*M*_, of 3.45 × 10^−4^ nm^2^ in the Co layer, corresponding to a bulk magnetization of 1,180 emu cm^−3^ (1 emu cm^−3^=1 kA m^−1^). Strikingly, the shape of the magnetic profile changes after conditioning in +40 V, with *ρ*_*M*_ reduced significantly—by 80%—at the GdO_x_/Co interface and 38% in the bulk. Subsequent conditioning at −40 V recovers the original shape and *ρ*_*M*_ increases to 92% of the as-grown value.

**Figure 2:**
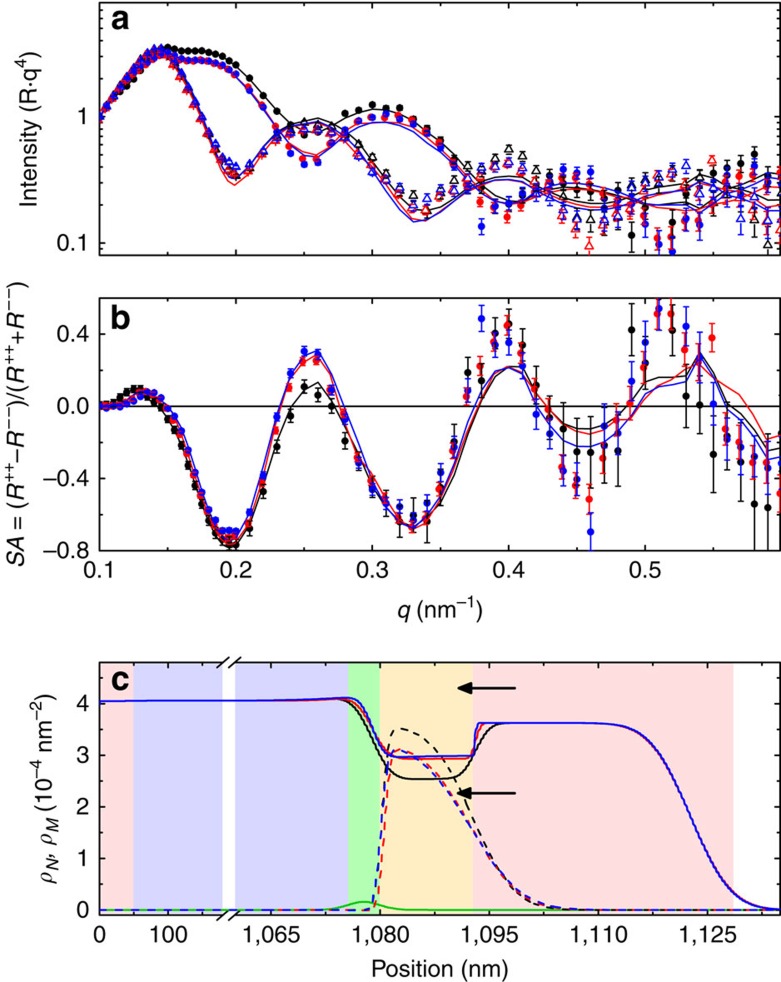
PNR results of thermally conditioned control sample. (**a**) Fitted PNR data, with R^++^ (R^*− −*^) identified by solid circles (open triangles), scaled by *q*^4^ and (**b**) spin asymmetry for the sample as-grown and after thermal-only conditioning. (**c**) Depth-dependent nuclear and magnetic SLD, *ρ*_*N*_ and *ρ*_*M*_, extracted from the PNR. In all panels black, red and blue lines identify the as-grown, once-treated (15 min) and twice-treated (2 × 15 min) samples, respectively. In **c** the solid and dashed lines identify *ρ*_*N*_ and *ρ*_*M*_, respectively, and the green line identifies *ρ*_*imag*_. Background colours in **c** represent (red) Pd, (purple) AlO_x_, (green) GdO_x_ and (yellow) Co. In **a** and **b** the experimental data are shown as symbols, and the lines are fits corresponding to the depth profile shown in **c**. Error bars in **a** and **b** correspond to ±1 s.d. Arrows in **a** and **b** identify the bulk *ρ*_*N*_ for Co (2.25 × 10^−4^ nm^−2^) and CoO (4.29 × 10^−4^ nm^−2^).

Control experiments were performed on a sample grown side-by-side with the E+T-conditioned sample following the same thermal treatment but without an electric field. PNR measurements of the as-grown sample, shown in Fig. 2, share a similar structure to the as-grown sample in Fig. 1, with small differences in *ρ*_*N*_ likely due to sample aging (30 days between measurements in Figs 1 and 2). Similar to the E+T sample, the first thermal treatment also increases *ρ*_*N*_ and reduces *ρ*_*M*_ in the Co layer, but to a much lesser degree and with no significant changes in the GdO_x_/Co interface shape and extent. Quantitative comparison shows that *ρ*_*N*_^*Co*^ increases by 17% and *ρ*_*M*_^*Co*^ decreases by 12% after thermal treatment alone compared with the as-grown sample. This is much less than the E+T-treated sample, which showed an increase in *ρ*_*N*_^*Co*^ of 34% and a decrease in *ρ*_*M*_^*Co*^ of 38% after the +40 V treatment. After a second 15-min thermal conditioning the nuclear and magnetic profiles of the control sample do not change appreciably, suggesting a saturation effect or depletion of easily diffusible oxygen. These results confirm the role of the electric field in enhancing the oxidation of the Co layer during the +40 V conditioning and reducing it during the −40 V treatment.

### Magnetometry

**Figure 3:**
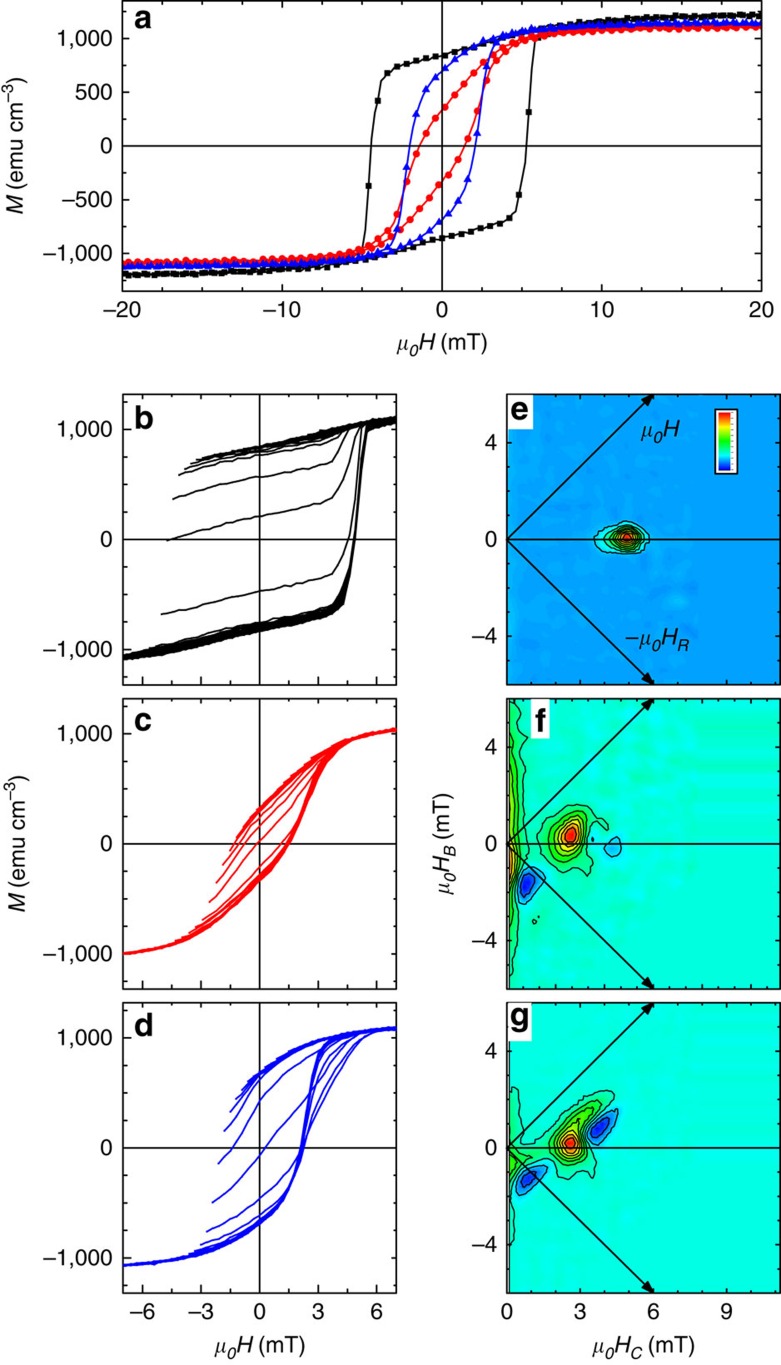
Magnetometry and FORC investigations. (**a**) Combined major hysteresis loops for the sample (black) as-grown, (red) after +/−40 V conditioning and (blue) after thermal-only conditioning. Family of FORCs and FORC distributions for the sample (**b**,**e**) as-grown, (**c**,**f**) after +/−40 V conditioning and (**d**,**g**) after thermal-only conditioning. Color bar in **e** identifies the max (red) and min (blue) value of the FORC distribution.

Magnetic hysteresis loops of the samples as-grown, after sequential +/−40 V treatment (E+T) and after two thermal-only treatments are shown in Fig. 3a. The Co *M*_*S*_ in the as-grown sample was measured to be 1,230 emu cm^−3^, in good agreement with the PNR value of 1,180 emu cm^−3^. Further, *M*_*S*_ decreased by 10% in the E+T-treated sample and 7% in the thermal-only sample compared with the as-grown sample, similar to the PNR data which show a reduction of 10%. The good agreement between the magnetometry and PNR results supports the validity of the model used to fit the data. Sample coercivity and remanent magnetization also decreased compared with the as-grown sample by 68% and 55% in the E+T sample and 54 and 11% in the thermal sample, respectively, indicating significant changes in the magnetic characteristics.

Details of the magnetization reversal have been investigated by the FORC method^26^,^27^,^28^,^29^. The family of FORCs and FORC distribution for the as-grown sample are shown in Fig. 3b and e, respectively. The family of FORCs show the minor loops fill the major loop, and the calculated FORC distribution shows only a single feature, centred at (local coercivity *μ*_*0*_*H*_*C*_=4.9 mT, bias field *μ*_*0*_*H*_*B*_=0 mT, where *μ*_*0*_ is the vacuum permeability 4π × 10^−7^ N A^−2^), indicating the sample is comprised of a single magnetic phase^29^,^30^. After the combined +/−40 V electric field treatment (E+T) the family of FORCs, Fig. 3c, still fill the major loop, but the FORC distribution, Fig. 3f, now shows two features. The main feature is centred at (*μ*_*0*_*H*_*C*_=2.6 mT, *μ*_*0*_*H*_*B*_=0.33 mT) and is circularly symmetric, again indicative of an irreversible (that is, hysteretic) phase. The shift in *H*_*C*_ relative to the as-grown sample indicates the coercivity is significantly reduced. The non-zero bias suggests a finite interaction experienced by this phase and may be the result of exchange bias with residual antiferromagnetic CoO with an enhanced Néel temperature^31^. A second phase is identified by the elongated FORC ridge along the *μ*_*0*_*H*_*B*_ axis centred at *μ*_*0*_*H*_*C*_=0 mT. This ridge represents a reversible phase with an internal demagnetizing interaction of 6 mT at saturation, identified by the spread of the feature along the *μ*_*0*_*H*_*B*_ axis^27^. A negative feature centred at (*μ*_*0*_*H*_*C*_=0.8 mT, *μ*_*0*_*H*_*B*_=−1.7 mT) identifies reversal events which are present on FORC branches that start at *H*_*R*_^1^, *M*(*H*,*H*_*R*_^*1*^), but absent on FORCs that begin at more negative *H*_*R*_^2^, *M*(*H*,*H*_*R*_^2^) with *H*_*R*_^2^<*H*_*R*_^1^ (ref. 29). In this case, the negative feature in Fig. 3f aligns in *H*_*R*_ with the peak of the reversible feature, and in *H* with the irreversible feature. This behaviour indicates that once the irreversible switching event occurs, the reversible phase changes its upswitching field due to magnetic coupling between the reversible and irreversible phases.

The family of FORCs for the thermally treated sample, Fig. 3d, is significantly different, with the minor loop protruding outside of the major loop. This indicates that the domain structure evolves more easily under fields applied along the major loop that increase from the saturated state, than under fields that increase from the mixed multi-domain state^32^. This result further underscores the role of the electric field in determining the oxygen distribution. Similar to the E+T sample, the FORC distribution for the thermal-only sample, Fig. 3g, also exhibits reversible and irreversible phases. The main FORC feature is centred at (*μ*_*o*_*H*_*C*_*=*2.6 mT, *μ*_*o*_*H*_*B*_=0.17 mT) and is no-longer circularly symmetric, but rather has a 90° bend with symmetries along the +*H* and -*H*_*R*_ axes, typical of a domain nucleation/growth reversal mechanism^28^,^33^. The FORC distribution for the thermal-only sample shows the same negative feature that again identifies magnetic coupling, and a new feature associated with the observed major loop protrusion. Integrating the FORC features gives a magnetic phase fraction^30^: the reversible phase contributes to 0%, 31% and 24% of the magnetization in the as-grown, E+T and thermal-only samples, respectively.

Reversible phases exhibit no hysteresis, and therefore are manifested in the FORC distribution along the *μ*_*o*_*H*_*C*_=0 mT axis, for example, when the phase has essentially zero coercivity or when the magnetic field is applied along the magnetic hard axis. Major hysteresis loops measured in the out-of-plane direction (see Supplementary Fig. 3 and Supplementary Note 2) for these samples display little hysteresis, implying that the out-of-plane direction remains the hard axis. These results suggest that oxygen migrates in the film after E+T or thermal-only conditioning and segregates to grain boundaries in the Co layer, thus disrupting long-range magnetic correlations and effectively breaking down the affected Co films into isolated grains. Once the coupling between the grains becomes weaker, their respective magnetocrystalline anisotropies in confined grains play a large role in determining the magnetic orientation resulting in much reduced coercivity and remanent magnetization.

### X-ray absorption and circular dichroism

**Figure 4:**
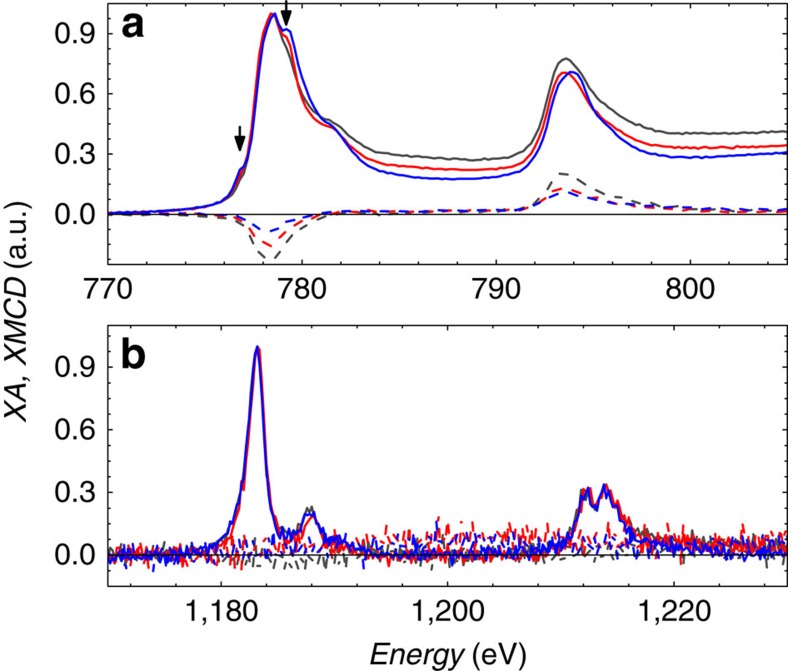
X-ray absorption and XMCD spectra. XA (solid) and XMCD (dashed) spectra for (**a**) cobalt and (**b**) gadolinium in samples as-grown (grey), after +/−40 V conditioning (E+T, red) and thermal-only (blue) conditioning. Arrows indicate 779.2 eV and 776.8 eV.

Oxidation of the cobalt after both +/−40 V E+T and thermal-only treatments is confirmed in the XA and XMCD measurements (see the ‘Methods' section) shown in Fig. 4a. Oxidation of the Co layer^34^ is identified directly by the emergence of peaks at *E*=779.2 eV and 776.8 eV, which are not present in the as-grown profile. The peak at 779.2 eV is largest in the thermal-only sample, indicating significantly increased oxidation relative to the E+T sample. This trend is supported by the XMCD spectra, which shows that the as-grown sample has the largest dichroism, indicating the largest magnetization. The E+T sample has the second largest dichroism, and the thermal-only sample has the smallest. The different ordering in the dichroism, compared with the bulk magnetometry, may identify variation in the depth-dependent oxygen-binding behaviour. This is consistent with the XA results, which showed a larger oxidation peak in the thermal-only treatment than the E+T sample. XMCD signal from the Gd, shown in Fig. 4b, shows no dichroism for all three samples, indicating a negligible contribution to the magnetization. Interestingly, the XA signal for the Gd shows no significant change for any of the samples, suggesting a relatively constant Gd oxidation state, regardless of oxygen migration into or out of the Co. While the Gd XAS is expected to be less sensitive to the oxidation state than the Co XAS, the amount of oxygen necessary to facilitate the observed changes in the Co would constitute a significant change if it had come from the 2-nm thick Gd layer, and would be observable in the Gd XAS.

The PNR, magnetometry and X-ray results clearly demonstrate that E+T conditioning can drive oxygen semi-reversibly into a thick (15 nm) Co film, profoundly changing its magnetic properties. Depth profiling with PNR indicates that while these effects are most prominent at the GdO/Co interface, they also extend throughout the entire 15-nm thick Co film. Reversing the polarity of the applied voltage drives oxygen out of the Co, partly restoring *ρ*_*N*_^*Co*^ and *ρ*_*M*_^*Co*^ to their original values at the GdO_x_/Co interface, but leaving *ρ*_*N*_^*Co*^ and *ρ*_*M*_^*Co*^ unchanged near the Co/Pd interface. Thermal conditioning of the control sample also promotes oxidation of the Co layer, but the supply of highly mobile oxygen that can be moved by entropy-driven diffusion is clearly limited. In the following discussion we determine the oxygen stoichiometry from the nuclear scattering profile and consider the underlying mechanics of the oxygen migration.

The role of the electric field and entropy-driven oxygen migration is seen qualitatively by comparing the profiles for the +40 V E+T-treated sample with the thermally treated sample (Figs 1c and 2c, respectively). The thermal treatment is shown to scale the magnetic depth profile relative to the as-grown sample, but not change its shape. In comparison, the magnetic depth profile for the +40 V sample strongly deviates from that in the as-grown sample, suggesting that the electric field drives oxygen into the film, while the thermally activated, entropy-driven oxygen migration is relatively uniformly distributed.

Using the neutron coherent scattering length, *b*, for cobalt (2.49 fm) and oxygen (5.81 fm) (ref. 35), the CoO_x_ stoichiometry can be directly calculated. Specifically, the nuclear SLD is calculated as: 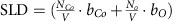 where *N*_*Co(O)*_ is the total number of cobalt (oxygen) atoms within the volume, *V*, of the Co film. Assuming the as-grown film is pristine Co, which is supported by the good agreement with the referenced bulk *ρ*_*N*_^*Co*^ value, and that the cobalt number density remains constant during treatment, a lower-limit to the oxygen profile can be calculated: 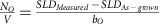. The stoichiometry is then defined as CoO_N(O)/N(Co)_. The nuclear depth profiles suggest that the +40 V conditioning forms CoO_0.18±0.01_, compared with CoO_0.07±0.01_ in the thermally treated sample. Treatment with a reversed polarity removes about one-third of the absorbed oxygen, leaving CoO_0.12±0.01_. The fact that the magnetization measurements show only a 10% reduction in *M*_*S*_ illustrates an indirect correspondence (for example, not one-to-one) of the magnetization and nuclear composition and suggests some of the O^2*−*^ may be forming magnetic compositions other than CoO, such as Co_2_O, or may remain as interstitial oxygen. Integrating the oxygen profiles for the conditioned samples shows conservation of oxygen within this system to within 3%. Since we do not expect any external sources of oxygen, this agreement is another good support for the validity of the presented models. With additional assumptions we can further determine a depth-resolved oxygen profile (see Supplementary Fig. 4 and Supplementary Note 3) to highlight the interface and bulk migration explicitly.

Interestingly, the depth profiles (especially the magnetic profile) indicate that the oxygen is semi-reversibly driven out of the GdO_x_/Co (0–10 nm) interface after treatment in −40 V, while the remaining oxygen is left trapped deeper within the Co. We suggest that as the GdO_x_/Co interface becomes depleted of oxygen, becoming more metallic. The oxidized region deeper within the film gets surrounded by conductive layers above and below, screening the electric field; without an electric field the oxygen does not migrate, resulting in the observed trapping effect. Thus, we suggest that the observed 10 nm thickness of the Co presents a practical limit for the electrically driven oxygen migration within these otherwise metallic films.

### Mechanics of oxygen migration

Brief considerations of the underlying mechanisms quickly reveal that this effect cannot be justified with bulk chemistry alone. First, considering the thermally treated sample, the initial treatment both suppresses the magnetism and increases the nuclear SLD, consistent with inclusion of oxygen. Further treatment only weakly changed these parameters, indicating that all of the easily diffusible oxygen had migrated during the first treatment. Since the available thermal energy (*k*_*B*_*T*=43 meV) is not enough to reduce Gd_2_O_3_ (enthalpy of formation, *ΔQ*=18.9 eV), Al_2_O_3_ (*ΔQ*=17.4 eV) or CoO (*ΔQ*=2.5 eV), we suggest the source of the mobile oxygen is likely interstitial, for example, from trapped sputtering gas located at grain boundaries or voids in the GdO_x_ and AlO_x_, which diffuses and reacts with the Co (Co+O→CoO, *ΔQ*=−2.5 eV or 2Co+O_2_→2CoO, *ΔQ*=−0.2 eV) (refs 36, 37).

Next, we consider the role of the electric field on oxygen mobility. Defect sites in oxygen-rich transition metal oxide films have been previously shown to act as p-type dopants^38^, giving them an effective negative charge. In the presence of an electric field with the anode on the Pd cap surface and cathode on the buried Pd seed film, excess O^2*−*^ defects in the GdO_x_ and AlO_x_ will be pulled towards the cobalt, while vacancies are pulled towards the bottom Pd electrode. The chemical potential within each of the AlO_x_, GdO_x_ and Co films is expected to be uniform, and thus O^2*−*^ defects are expected to be highly mobile within each^17^. The application of a static electric field then causes field-induced ion migration^39^. However, at the boundary between two layers there will exist a difference in the chemical potential, which may cause an accumulation of oxygen at, for example, the GdO_x_/Co interface. Considering this issue, the enthalpy of formation is calculated in each layer and compared with the electric potential energy available to overcome this interfacial barrier. Assuming a lattice-site hopping model^40^,^41^, the electric potential energy at the interface can be calculated to be 24 meV (*qEΔx*, where *q* is the oxygen charge of 2e^*−*^, *Δx* is the atomic site spacing of 3 Å and *E* is the electric field of 400 kV cm^*−*1^). The sum of the electric potential energy and thermal energy defines the scale of the energy landscape available to drive the reversible oxygen migration back and forth across the interface. First considerations of an ideal system were that the migrating oxygen is moved from chemically stable Gd_2_O_3_ to the Co (Gd_2_O_3_+3Co→3CoO+2Gd, *ΔQ*=+11.4 eV) (refs 36, 37). Clearly the available energies (67 meV) cannot drive this reaction; stability of the Gd_2_O_3_ oxidation state is supported by the XA in Fig. 4. An alternative scenario suggested above is that the trapped oxygen react with the Co to form CoO (2Co+O_2_→2CoO, *ΔQ*=−0.2 eV). However, without a charge, the O_2_ experiences no net force from the electric field, suggesting this reaction is not responsible for the observed effects induced by the electric field. Similar reactions with elemental oxygen (Co+O→CoO, *ΔQ*=−2.5 eV) can occur along the forward reaction, but will not be reversible as it again costs too much energy. Other considered reactions are presented in Supplementary Note 4, but no bulk energy calculation was able to support the observed results.

**Figure 5:**
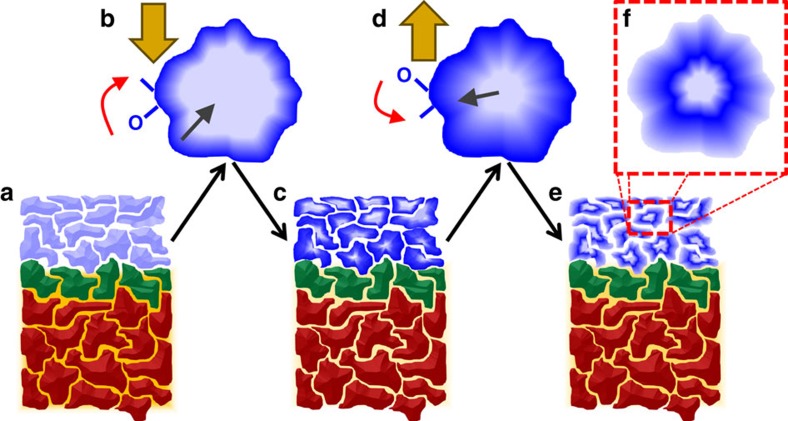
Illustration of oxygen migration mechanism. Cross-section view of (**a**) the as-grown film, (**b**) a single grain during +40 V treatment, (**c**) the film after +40 V treatment, (**d**) a single grain during subsequent −40 V treatment, and (**e**, **f**) cross section and grain after −40 V treatment. Colours identify AlO_x_ (red), GdO_x_ (green), metallic FM Co (light blue), insulating non-FM CoO_x_ (blue) and interstitial oxygen (orange); large gold arrow indicates the electric field. Illustration emphasizes fast surface migration, identified by the red arrows, and slow bulk diffusion, indicated by the grey arrows.

We present a possible alternative energy consideration, treating the grains as nanoscale clusters, which provides a reasonable energy landscape for the observed migration. In the picture illustrated in Fig. 5, oxygen ions are bound to the surface of the nanocrystalline grains. These O^2*−*^ ions are off-stoichiometry defects, and have a different binding energy than the core of the grain. Since the grains were all fabricated at the same time by sputtering in an oxygen-rich environment, the surface structure and hence energy landscape, is expected to be relatively uniform. Thus applying an electric field moves the surface O^2*−*^ towards the anode and into the Co layer, forming CoO_x_. Once on the grain surfaces these O^2*−*^ ions experience chemically driven diffusion, which occurs at a rate of ∼10–20 nm h^*−*1^ at these temperatures^42^, preferentially oxidizing the surface, but likely also the core of the small cluster-like Co grains. Reversing the applied field drives O^2*−*^ to leave the CoO_x_ film. Using a cluster-like formation approach, the binding energy per atom for GdO_x_ (ref. 43) and CoO_x_ (ref. 44) can be determined by modelling to be 4.50 and 4.48 eV per atom near nominal stoichiometry, respectively. Since the energy landscape is isotropic to within 20 meV, one only has to overcome the small barrier and the activation energy to move oxygen ions, destroy old bonds and create new ones. Thus oxygen freely leaves the CoO_x_, but due to the slow bulk diffusion in the CoO_x_ grains, a gradient oxygen profile is expected to form within the grains, with the surface being oxygen deficient. Once the grain surface loses enough oxygen to become conductive, the internal electric field is again screened, trapping the oxygen within the core. Thus, both at the film-length mesoscale and grain-length nanoscale, the oxygen migration induces screening effects which limits the oxygen mobility. We propose that, as a result, the conditioned films have a highly defective structure, with a complex mix of CoO_x_ core-shell-surface grains. This model presents a mechanism which is semi-reversible and nearly recovers the saturation magnetization, thus consistent with the PNR and magnetometry. Perhaps more remarkably, this model also has a disrupted long-range ordering, consistent with the two-phase construction identified by the FORC measurement. Finally, this model also agrees well with the XA spectra; the oxygen migration in the Co grains occurs both on the surface and throughout the bulk. In contrast, only the oxygen on the surface of the GdO_x_ grains is likely mobile with the bulk of the grain remaining stoichiometry balanced.

In summary, we report the first direct depth profile mapping of voltage-moderated oxygen migration in magnetic Co thin films. Using X-ray spectroscopy and PNR we observed changes in the structural and magnetic profiles, consistent with partial oxidation of the Co layer. The oxidation and corresponding magnetic changes associated with application of a +40 V treatment were found to be strongest at the GdO_x_/Co interface. We showed that the interfacial oxidation was largely reversible, and could be driven back to the Co layer with a reversed electric field, while the oxygen deeper in the film remained trapped. The effects of the electric field and thermal treatments were separated by comparing samples conditioned with and without an electric field. Magnetometry using the FORC technique revealed that the treatment altered the magnetic properties of the film, resulting in two distinct magnetic phases. X-ray spectroscopy revealed increased oxidation in the Co film after any conditioning, but less after the E+T treatment with the reversed polarity. Thermal and electric cycling markedly change the granular structure of the system, providing a means by which the GdO_x_ can easily transport the oxygen. These results provide a depth-resolved view of magneto-ionic motion beyond the conventional interface limit, opening a new avenue to explore their applications in future device concepts.

## Methods

### Sample fabrication

Thin film samples with a structure of Pd (40 nm)/AlO_x_ (1 μm) were fabricated by e-beam evaporation on naturally oxidized Si substrates in a high vacuum (base pressure of 10^*−*4^ Pa) chamber. Deposition was performed at 150 °C from nominally Al_2_O_3_ beads and 99.9% Pd ingots. This thick layer was necessary to ensure a pinhole-free electrically insulating layer over the sample area (∼1 cm^2^). Next, a 2-nm GdO_x_ film was deposited by reactive DC sputtering (in another chamber with base pressure of 10^*−*4^ Pa) in a 0.7 Pa O_2_:Ar (1:3) atmosphere. During deposition of the AlO_x_ and GdO_x_, an edge was covered to allow for electrical contact to the Pd layer. Finally, using a contact shadow mask, 1-cm^2^ films of Co (15 nm)/Pd cap (20 nm) were deposited by DC sputtering in a 0.5-Pa Ar atmosphere. Using a shadow mask gives the film an oxide window frame which prevents edge-shorting between the top and bottom Pd contacts. Electrical connections were made to the top and bottom Pd films by silver paint; the resistance through the thickness of the sample was measured to be >10 MΩ indicating minimal contributions from pinholes, if any.

### Characterizations

The sample was characterized in the as-grown state, then E+T conditioned by heating it to 230 °C and applying a voltage of +40 V (400 kV cm^*−*1^)—with the anode on the top surface—for 15 min. After measuring the sample in the conditioned state the sample was again heated to 230 °C with a voltage of −40 V applied for 15 min. Control measurements were performed on identical films (grown side-by-side) where only heating was performed, hereafter referred to as thermally treated (or thermal-only).

PNR measurements^45^ were performed at the NIST Center for Neutron Research (NCNR) on the MAGIK and PBR reflectometers at room temperature with an in-plane applied magnetic field of 17 mT. Incident (scattered) neutrons were polarized with their spin parallel (+) or antiparallel (−) to the field, and the non-spin-flip specular reflectivities (*R*^*Incident scattered*^: *R*^*++*^ and *R*^*−−*^) were measured as a function of wave vector transfer *q*. Magnetometry results measured at room temperature on a vibrating sample magnetometer show that the applied 17 mT field is large enough to saturate the cobalt magnetization. Thus the spin-flip reflectivities (*R*^*+−*^ and *R*^*−+*^) are zero, and are not considered here. The spin asymmetry representation of the data is calculated as *SA*=(*R*^*++*^−*R*^*−−*^)/(*R*^*++*^+*R*^*−−*^). Within the Born approximation, the numerator of the *SA* is proportional to the product of the magnetization and nuclear SLD; for non-magnetic samples *R*^*++*^=*R*^*−−*^, thus *SA*=0. Although it is a non-trivial mixture of magnetic and structural scattering, the *SA* can give a qualitative sense of the sample's magnetism. Model fitting of *R*^*++*^(*q*) and *R*^*−−*^(*q*) allows for determination of depth (*z*)-dependent nuclear and magnetic SLD^46^,^47^. Model fitting of the PNR data was performed using the Refl1D software package^48^ and error bars determined by using a Markov chain Monte Carlo method using the BUMPS software package^49^. In the model the Si, bottom Pd seed and AlO_x_ are slabs with a uniform SLD and no magnetic contribution. The GdO_x_, Co and top Pd cap are modelled as a sum of error-function interfaces with fitted locations, widths and heights. A second model for the uncoated window frame was included as an incoherent sum. The data for the as-grown and conditioned states were simultaneously fitted with identical parameters for the Si, Pd seed and AlO_x_ shared between the models. The nuclear SLD, *ρ*_*N*_, presented in the main text are the mean-values, but appear in the fitted profile as a weighted average with neighbouring layers at the interface. The neutron is sensitive to the net scattering potential at a defined depth, thus surface roughness and conformal roughness act to average the SLD at the interfaces.

FORC measurements were performed following previously outlined measurement procedures^26^,^29^,^30^,^50^,^51^. From positive saturation the magnetic field is decreased to a scheduled reversal field, *H*_*R*_, then the magnetization, *M*, is measured as the applied field, *H*, is increased from *H*_*R*_ back to positive saturation. This measurement process is repeated for a range of *H*_*R*_ between positive and negative saturation, collecting a family of FORCs which fill the interior of the major loop. A mixed second order derivative is applied to extract the FORC distribution: 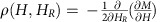. To capture the reversible magnetic behaviour, a constant extension is applied to the *H=H*_*R*_ boundary of the data set: *M(H<H*_*R*_*)≡M(H*_*R*_) (ref. 27). Recognizing that progressing to more negative values of *H*_*R*_ probes downswitching events, and increasing *H* probes upswitching, a new coordinate system is defined in terms of a local coercivity and bias field: 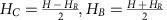, respectively.

XA and XMCD measurements were performed at the Advanced Light Source on beamline 4.0.2. Elemental sensitivity was achieved by probing the Co *L*_*2,3*_ edges, and Gd *M*_*4,5*_ edge, following previously outlined procedures^52^,^53^,^54^. Measurements were performed using a constant beam polarization and an alternating in-plane magnetic field of ±200 mT. Signal was detected by fluorescence yield.

### Data availability

The data that support the findings of this study are available from the corresponding authors on request.

## Additional information

**How to cite this article:** Gilbert, D. A. *et al*. Structural and magnetic depth profiles of magneto-ionic heterostructures beyond the interface limit. *Nat. Commun.* 7:12264 doi: 10.1038/ncomms12264 (2016).

## Supplementary Material

Supplementary Information Supplementary Figures 1-4, Supplementary Notes 1-4 and Supplementary References
