# Supplementary material for: Structural and magnetic depth profiles of magneto-ionic heterostructures beyond the interface limit
Source: Nat Commun. 2016 Jul 22;7:12264. doi: 10.1038/ncomms12264 (PMC4961844; doi:10.1038/ncomms12264)
Supplement: Supplementary Information — Supplementary Figures 1-4, Supplementary Notes 1-4 and Supplementary References [file ncomms12264-s1.pdf]

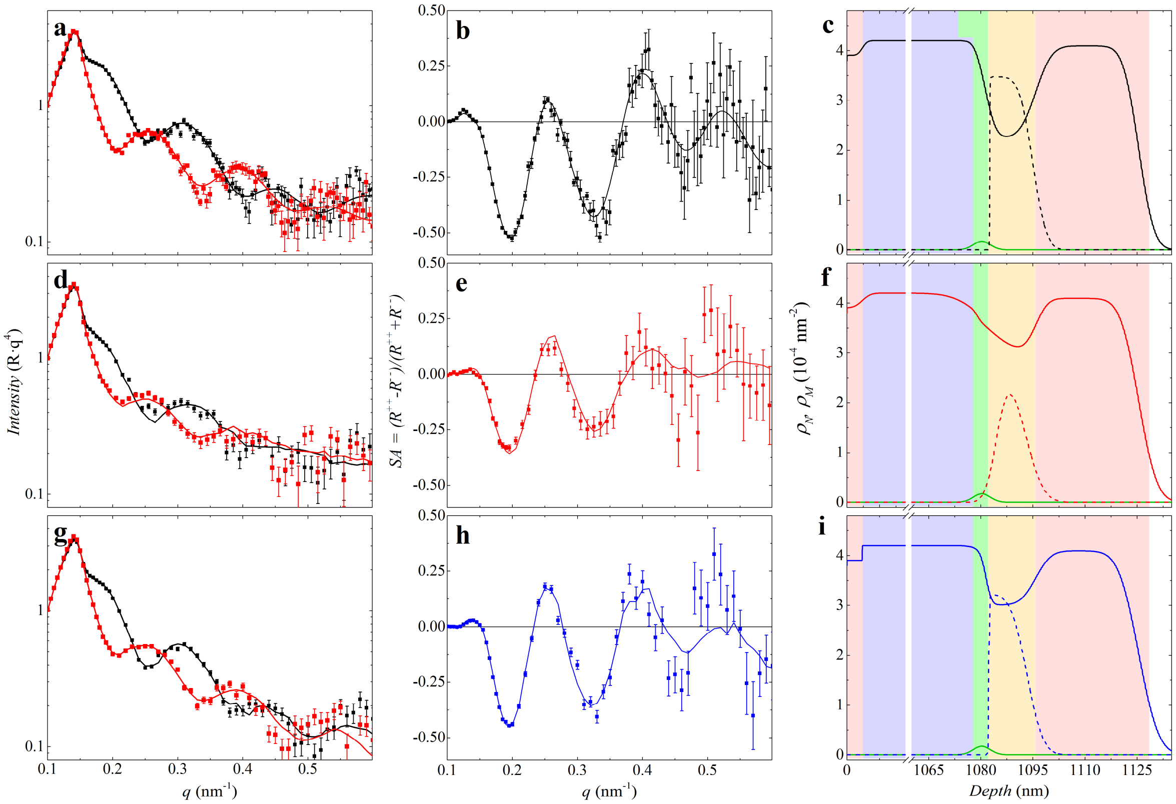

Supplementary Figure 1. PNR results from the Electro-Thermal Treatment in Individual Panels. (**a, d, g**) Fitted PNR data scaled by q^4 and (**b, e, h**) spin asymmetry and (**c, f, i**) fitted depth dependent real and imaginary nuclear SLD (ρ_N and ρ_{imag}), and magnetic SLD (ρ_M) for the GdO_x/Co sample (**a-c**) as-grown, (**d-f**) after +40 V treatment, and (**g-i**) after sequential +/- 40 V treatment. In (**a, d, g**) the R^{++} (R^{--}) channel is shown in black (red). In (**c**, **f**, **i**) solid lines show ρ_N, dashed line show ρ_M, green line shows $\rho_{imag.}$. Error bars in (**a, d, g**) and (**b, e, h**) identify one standard deviation.

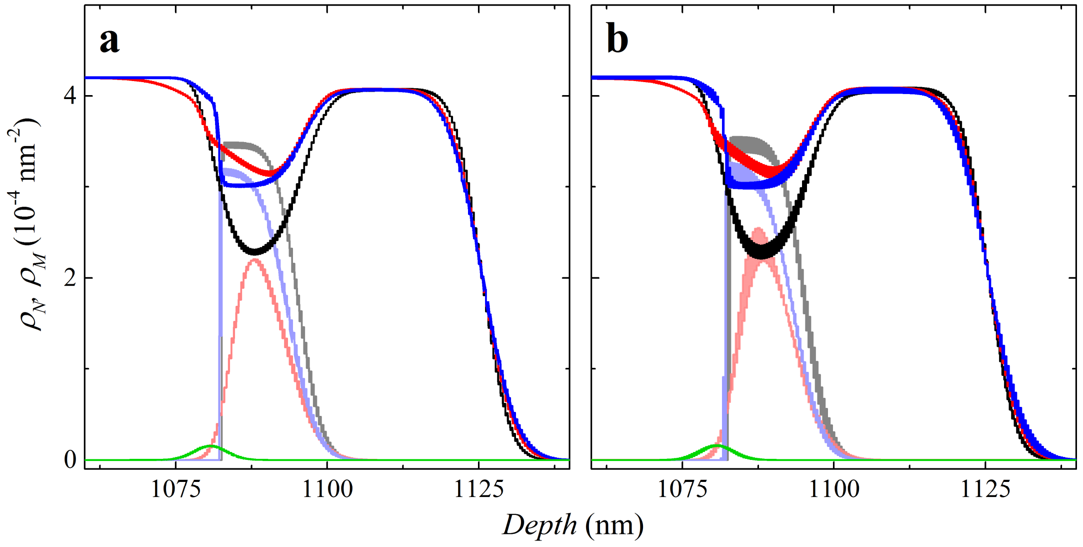

Supplementary Figure 2. **Converged Depth Profiles with Error Bar Envelopes.** Main text Figure 1c with error bar envelopes of (a) 68% confidence interval (one-sigma) and (b) 95% confidence interval. Plots show ρ_N (bright), ρ_M (pastel), and $\rho_{imag.}$ (green) for the (black) as-grown, (red) after +40 V conditioning and (blue) after +/- 40 V conditioning.

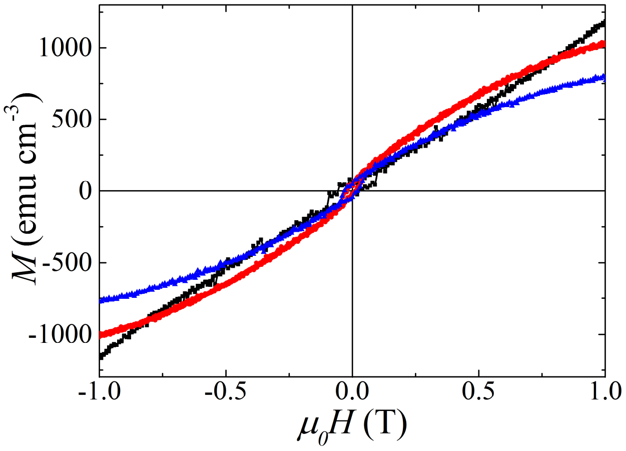

Supplementary Figure 3. **Out-of-Plane Magnetometry.** Out-of-plane hysteresis loops for the samples as-grown (black), after sequential +/- 40 V (E+T, red) and thermal-only treatments (blue). (1 emu cm^{-3} = 1 kA m^{-1})

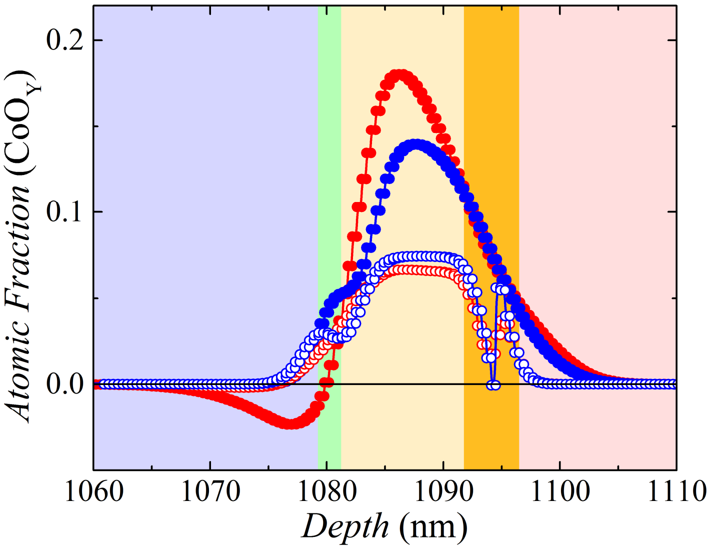

Supplementary Figure 4. **Calculated Oxygen Depth Profile.** Calculated depth-resolved oxygen stoichiometry for the (solid) electro-thermally and (open) thermal-only conditioned samples, after their first (red) and second (blue) treatments. As in the Main Text Fig. 1c, colors represent (red) Pd, (purple) AlO_x, (green) GdO_x, (light yellow) first 10 nm of Co, and (dark yellow) top 5 nm of Co respectively.

Supplementary Note 1

A universal issue with fitting neutron data is the uniqueness of the fit. That is, for a given data set there may be multiple profiles which reproduce the experimentally measured scattering pattern. Note that the fitted profile is determined from the whole reflectometry pattern, and as such is more sensitive to trends in the data than single-point variation. We have a high-level of confidence in our fits because (1) they reproduce approximately the accepted bulk values of nuclear and magnetic SLD for the constituent elements, (2) the measured structure agrees well with the designed structure, and (3) for a single sample the fits following 3 different treatments

were performed in parallel where some fitting parameters (e.g., film thickness, SLD of the bulk of the AlO_x and substrate) are coupled. Confidence factor (3) means that any model would not only have to reproduce one scattering pattern, but all three.

Error bars were determined for the converged fit using a Markov chain Monte-Carlo method and plotted in Supplementary Figure 2. Supplementary Figure 2a shows the converged best fit with a 1-sigma (one standard deviation, e.g. 68% confidence) envelope; Supplementary Figure 2b shows the converged best fit with a 95% confidence envelope. In both cases we note that the changes in magnetic and nuclear SLD, as well as the interface widths are still well resolved.

To further demonstrate confidence in the presented model, we attempted to fit the data with models having fixed magnetic and nuclear profiles. That is, the nuclear (magnetic) profile is constrained to be constant throughout all of the models, probing whether the PNR results can be accounted for by only magnetic (nuclear) changes. In both cases the models would not converge to any reasonable value (e.g. physically unreasonable nuclear SLDs and interface widths, and thicknesses significantly different from the designed structure).

Supplementary Note 2

As noted in the main text, the 15 nm Co film is expected to have an in-plane easy axis. Out-of-plane major hysteresis loops for the samples as-grown, after sequential +/-40 V and thermal-only treatments are shown in Supplementary Figure 3. The plot shows no significant hysteresis, and exceedingly small remanence and coercivity for all samples. This behavior identifies that the out-of-plane direction is the magnetic hard axis, as expected from the shape anisotropy.

Supplementary Note 3

In the main text, the oxygen content was calculated from the fitted SLD of the Co layer and the nuclear scattering lengths of Co and O. Two assumptions were made: (1) the as-grown Co-film was pristine, and (2) the number of Co atoms in the layer volume remained constant. Both of these are expected to be accurate based on the fitted SLD of the as-grown film and the fitted thicknesses. Expanding on (2) and assuming that the Co atoms do not migrate within the layer, the same approach can be used to calculate the depth-resolved oxygen profile, shown in Supplementary Figure 4. This plot shows explicitly the separate effects at the bottom 10 nm of the Co film (starting at approximately 1083 nm), near the GdO_x/Co interface, and at the top 5 nm, near the Co/Pd interface. The thermal-only samples confirm oxidation after the first treatment and little additional oxidation after the second. This plot also shows the large difference between the electro-thermally treated samples and the thermal-only sample.

Supplementary Note 4

Below is a list of potential enthalpy of reactions for bulk gadolinium, cobalt and oxygen, with the calculated enthalpy of reaction.[1, 2] Positive enthalpy of reaction indicate the reaction is endothermic and thus must consume at least that much energy from the system. Negative enthalpy of reaction indicates exothermic. In order to be consistent with the observed results - including reversibility in the interfacial cobalt oxidation, by reversing the electric field - the enthalpy must be on a similar scale to the thermal + electric potential energy (67 meV).

$$\mathrm{GdO+Co} \rightarrow \mathrm{CoO+Gd},\ \Delta Q = -1.8\ \mathrm{eV}$$

$$Gd_2O_3+3Co \rightarrow 3CoO+2Gd, \Delta Q = +11.4 \text{ eV}$$

$$Gd_2O_3+Co \rightarrow CoO+2GdO, \Delta Q = +15 \text{ eV}$$

$$3GdO+2Co \rightarrow Co_2O_3+3Gd, \Delta Q = -5.3 \text{ eV}$$

$$Gd_2O_3+2Co \rightarrow Co_2O_3+2Gd, \Delta Q = +15.7 \text{ eV}$$

$$4GdO+3Co \rightarrow Co_3O_4+4Gd, \Delta Q = -5.1 \text{ eV}$$

$$4Gd_2O_3+9Co \rightarrow 3Co_3O_4+8Gd, \Delta Q = +52 \text{ eV}$$

All the above reactions - in addition to the ones presented in the paper - suggest that the chemical energies are much larger than the thermal and electric potential energy.

References

1. Haynes WM. CRC Handbook of Chemistry and Physics. 93rd edn. CRC Press (2012).
2. Konings RJM*, et al.* The Thermodynamic Properties of the f-Elements and their Compounds. Part 2. The Lanthanide and Actinide Oxides. *J Phys Chem Ref Data* **43**, 013101 (2014).
